# Identification and characterization of epizootic hemorrhagic disease virus serotype 6 in cattle co-infected with bluetongue virus in Trinidad, West Indies

**DOI:** 10.1016/j.vetmic.2018.12.009

**Published:** 2019-02

**Authors:** Tamiko Brown-Joseph, Paulina Rajko-Nenow, Hayley Hicks, Nikita Sahadeo, Lara E. Harrup, Christine V. Carrington, Carrie Batten, Christopher A.L. Oura

**Affiliations:** a Department of Pre-Clinical Sciences, Faculty of Medical Sciences, The University of the West Indies, St. Augustine, Trinidad, West Indies; b Non-vesicular reference laboratory, The Pirbright Institute, Woking, Surrey, GU24 0NF, UK; c Entomology Group, The Pirbright Institute, Woking, Surrey, GU24 0NF, UK; d School of Veterinary Medicine, Faculty of Medical Sciences, The University of theWest Indies, St. Augustine, Trinidad, West Indies

**Keywords:** EHDV serotypes, Trinidad, cattle, EHDV evolution rate, BTV co-circulation, *Culicoides*

## Abstract

Epizootic hemorrhagic disease virus (EHDV) is an economically important virus that can cause severe clinical disease in deer and to a lesser extent cattle. This study set out to determine and characterize which EHDV serotypes were circulating in Trinidad. Serum and whole blood samples were collected monthly for six months from a cohort of cattle imported to Trinidad from the USA. Results revealed that all the cattle seroconverted to EHDV within six months of their arrival, with EHDV RNA being detected in the samples just prior to antibodies, as expected. Serotyping assays revealed that a single serotype (EHDV-6) was circulating in the cattle. Sequencing of the surface viral protein (VP2) of EHDV-6, followed by phylogenetic analysis, revealed that the Trinidad EHDV-6 strain was closely related to EHDV-6 viruses found in Guadeloupe (2010), Martinique (2010) and USA (2006), with 96–97.2% nucleotide identity. The Trinidad EHDV-6 VP-2 shared 97.2% identity with the Australian EHDV-6 prototype strain, classifying it within the eastern topotype clade. Bayesian coalescent analysis support Australia as the most probable source for the EHDV-6 VP2 sequences in the Americas and Caribbean region and suggests that the they diverged from the Australian prototype strain around 1966 (95% HPD 1941–1979).

## Highlights

• Epizootic haemorrhagic disease virus serotype 6 (EHDV-6) is circulating in Trinidad.
• EHDV is infecting cattle at a slower rate than BTV.
• EHDV appears to have a faster viral evolution rate than BTV.
• The EHDV-6 Trinidad strain (VP-2) falls within the eastern topotype clade that is likely to have originated from Australia.

## 1 Introduction

Epizootic Haemorrhagic Disease Virus (EHDV) and bluetongue virus (BTV) are separate species within the genus *Orbivirus* and the family *Reoviridae*. EHDV is closely related to BTV, both being double-stranded RNA viruses with ten segments that code for eleven distinct viral proteins (VPs) (Mecham & Dean, 1988). Seven of these proteins are structural (VP1-VP7) and arranged in three concentric shells to house the viral genome, and three of the proteins code for four non-structural proteins (NS1, NS2, NS3a and NS3b) (Anthony et al., 2009). Despite showing clinical similarities, these two viruses are genetically distinct and do not cross-react serologically. Both viruses are transmitted by the *Culicoides* biting midge (Diptera, Ceratopogonidae), with different midge species present in diverse geographical locations around the world having different levels of competence for the onward transmission of each virus (Aradaib & Ali, 2004; Federici et al., 2016). EHDV is responsible for the highly infectious, yet non-contagious, epizootic haemorrhagic disease (EHD), which was first described in 1955 in a New Jersey (USA) outbreak, White-tailed deer (Odocoilius virginianus (Zimmermann) (Shope et al., 1960) and other cervid species are most severely affected by the virus, often resulting in high levels of mortality associated with high fever, lethargy, oedema, ulcerations of the dental pad and oral mucosa, haemorrhaging of the heart, lungs, major blood vessels and other tissues. Less severe or asymptomatic (EHDV) infections are usually observed in cattle, which are considered to be the reservoir host for the virus (Maclachlan et al., 2015; Gibbs and Lawman, 1977). However, there have been reports of mild to severe clinical outbreaks of EHD in cattle in Japan (EHDV-2), Réunion Island (EHDV-1, 2, 3 and 6), Israel (EHDV-7), Morocco, Algeria, Tunisia and Turkey (EHDV-6) (Mejri et al., 2018; Cêtre-Sossah et al., 2014; Allison et al., 2010; Temizel et al., 2009; Anthony et al., 2009; Yadin et al., 2008; Gaydos et al., 2004; Bréard et al., 2004). Many of these outbreaks of EHD have resulted in serious economic losses (Kedmi et al., 2010).

The outer capsid protein VP2 of EHDV is a target for the protective immune response generated by the mammalian host. VP2 contains the majority of epitopes that are recognized by neutralizing antibodies and is therefore also the primary determinant of EHDV serotype. EHDV is currently classified into seven serotypes (1, 2, 4, 5, 6, 7 and 8). Serotype 3 is now considered as a strain of EHDV-1; EHDV-318 (also referred to as EHDV-9) is now considered a strain of EHDV-6 (Anthony et al., 2009) and the Ibaraki virus, first identified in cattle in Japan in 1959, is now considered a strain of EHDV-2 (Uchinuno et al., 2003).

EHDV- 1 and 6 are endemic throughout the USA in both wild and domesticated ruminants, while EHDV-2 is primarily endemic in south-eastern USA (Murphy et al., 2005) and is the most commonly detected EHDV serotype infecting White-tailed deer in the USA (Sun et al., 2014). In the fall of 2006, a novel reassortant EHDV strain was isolated in Indiana, USA, where the outer capsid genes (coding for VP2 and VP5) were from the exotic EHDV-6 Australian prototype strain (CSIRO 753), which was first isolated from sentinel cattle in the Northern Territories, Australia in 1981 (St. George et al., 1983), and the remaining gene segments coding for both non-structural (NS1 and NS3) and structural (VP1, VP3 and VP7) proteins were from the endemic EHDV-2 Alberta strain (Allison et al., 2010; Allison et al., 2012).

Although EHDV- 1, 2 and 6 are endemic in various areas of North America, South America and the Caribbean Basin, no clinical outbreaks in cattle had been reported (Verdezoto et al., 2017; Viarouge et al., 2014; Anbalagan & Hause, 2014; Allison et al., 2010) until 2013, when EHD was reported in cattle from Illinois, USA following an outbreak in deer in the same location in 2012 (Stevens et al., 2015). Viral antibodies were confirmed to be present in the cattle, but the EHDV serotype was not identified (Garrett et al., 2015). Previous serological studies identified EHDV- 1 and 2 to be circulating in the Caribbean Basin and South America (Gumm et al., 1984) and more recent studies have shown the presence of EHDV-6 in the Caribbean islands of Guadeloupe and Martinique and in French Guiana on the South American mainland (Viarouge et al., 2014). Additionally, EHDV-1 and EHDV-6 were recently identified in asymptomatic cattle in Ecuador (Verdezoto et al., 2016).

The aims of this study were to identify which of the seven EHDV serotypes are currently circulating in Trinidad and to characterize and compare the identified strains with others that are circulating in the region and globally.

## 2 Materials and methods

### 2.1 Cattle

In June 2013, sixty dairy cattle (5 bulls and 55 pregnant cows) were imported into Trinidad from the USA to improve genetic stock. Twenty-two of the cattle were Holstein, 37 were Jersey and one (bull) was Aberdeen Angus. The cattle were bled three days after their arrival in Trinidad (month 0) and then monthly for six months (months 1 to 6).

### 2.2 EHDV serology

Serum samples were tested for EHDV-speciﬁc antibodies using the LSI Ruminant ELISA kit REHDV (Invitrogen, ThermoFisher Scientific, Paisley, UK), an EHDV group-specific competitive ELISA (PrioCHECK™ EHDV Ab Serum Kit; Applied Biosystems ThermoFisher Scientific, Paisley, UK), following manufacturer’s instructions.

### 2.3 RNA extraction and EHDV group-specific real-time reverse transcription polymerase chain reaction (rRT-PCR)

Viral RNA was extracted from EDTA whole blood samples using MagVet Universal Isolation Kits (ThermoFisher Scientiﬁc, Paisley, UK) with the KingFisher Flex Extraction System (ThermoFisher Scientiﬁc, Paisley, UK). The extracted RNA was amplified with a group-speciﬁc rRT-PCR (Maan et al., 2017) using an Applied Biosystems 7500 Real-Time PCR System (ThermoFisher Scientiﬁc, Paisley, UK).

### 2.4 EHDV serotyping by conventional RT-PCR and DNA sequencing

Selected (first-time) positive EHDV samples (n = 22) with Ct values ranging from 22.3 to 35.4 (average Ct value 28.5) from the group-specific rRT-PCR were serotyped using a conventional RT-PCR assay previously described by Sailleau et al. (2012). The genetic sequence for genome segment-2 phylogenetically clusters the seven EHDV serotypes into four subgroups (Group A: EHDV-2 and EHDV-7; Group B: EHDV-4 and EHDV-5; Group C: EHDV-6 and EHDV-8; Group D: EHDV-1) (Anthony et al., 2009). Each RNA sample underwent four conventional RT-PCRs in duplicate using primers designed for each of the EHDV subgroups (A–D). The amplified products (10 μl) were subsequently analysed on 1.5% (*w/*v) agarose gels. Positive reactions were identified by the presence of bands of the expected size and the corresponding PCR product was sent to a commercial facility for DNA sequencing (Macrogen Inc., Seoul, South Korea). The sequences were then compared with related sequences in GenBank to identify the EHDV serotype.

### 2.5 EHDV serotyping using serotype-specific rRT-PCRs

Selected EHDV-positive samples (n = 14) were also tested by EHDV serotype-speciﬁc rRT-PCRs using available primers for the eastern (e) and western (w) EHDV topotypes 1e, 1 w, 2e, 2 w, 4 w, 5 w, 6e, 7e, 7 w, 8e and 9 w held at The Pirbright Institute (Surrey, UK) (Anthony et al., 2009).

### 2.6 Virus isolation

Blood samples (n = 20, with Ct values <30) from the EHDV group-speciﬁc rRT-PCR assay were selected for viral isolation in KC cells (derived from embryonic *Culicoides sonorensis* Wirth and Jones 1957 (Wechsler et al., 1991) as previously described (Batten et al., 2011). Seven days after inoculation, cell supernatant was tested by rRT-PCR for the presence of EHDV RNA. Positive cultures were stored at −80 °C.

### 2.7 Sequencing and phylogenetic analysis of the complete Trinidad EHDV segment 2 (VP2) gene sequence

The EHDV segment 2 (full length) of the Trinidad isolate was sequenced at The Pirbright Institute (Surrey, UK). Briefly, total RNA was extracted from cell culture pellet using TRIzol Reagent (ThermoFisher Scientiﬁc, Paisley, UK)and ssRNA was removed by precipitation with 2 M LiCl (Sigma, UK) at 4 °C for 24 h described previously (Maan et al., 2017). Libraries were prepared using the Nextera XT library preparation kit and sequencing was performed using MiSeq instrument. Pre-alignment quality check was performed using the FASTQC programme and sickle programme was used to trim reads at the quality threshold of 30. Reads were aligned to the reference genome (EHDV-6/CSIRO 753, segment 2, AM745038) using the BWA-MEM tool; and a combination of samtools and bcftools used to derive the consensus sequence (GenBank accession number MH446371, length 2971bp).

Related EHDV segment 2 sequences identified by BLAST searching on Genbank (Madden, 2003) were downloaded and aligned with the Trinidad EHDV VP2 sequence using the ClustalW algorithm in the Geneious v.9.04 software package. A pairwise distance matrix was generated and duplicate sequences were removed resulting in a final data set of 52 sequences (2910 to 2961bp in length). Shorter sequences were deleted and the remaining sequences were realigned to a consensus sequence length of 3004bp.

To discern the phylogenetic relationship of the Trinidad EHDV isolate among the global EHDV segment 2 sequences, a maximum likelihood phylogenetic tree was inferred from the data set using PhyML (Guindon et al., 2010) under the best fit model (general time reversible substitution model with a discrete gamma distribution with 4 rate categories and invariant sites, GTR + G_4_+I) as determined using JMODELTEST programme (Darriba et al., 2012). The reliability of the phylogenetic tree was evaluated using bootstrap testing (500 replicates). A subset of 16 EHDV-6 VP2 sequences was aligned with the Trinidad EHDV VP2 sequence as described above and trimmed to a common length of 2916bp. Substitution rates, times to the most recent common ancestor (T_MRCA_) and ancestral location states were jointly estimated using BEAST version 1.83, with the GTR + G_4_+I model of nucleotide substitution, along with a relaxed lognormal molecular clock model and the best-fit logistic population growth model. Analyses were run in duplicate for 90 million generations each and combined using Log Combiner version 1.8.3 with 5% removed as burn-in, and convergence of parameters was assessed by calculating the effective sample size (ESS > 100) using TRACER version 1.6. Table S1 shows the accession numbers, countries of origin and years of isolation of all sequences included in this study.

## 3 Results

### 3.1 All naïve cattle seroconverted by month 6

**Fig. 1:**
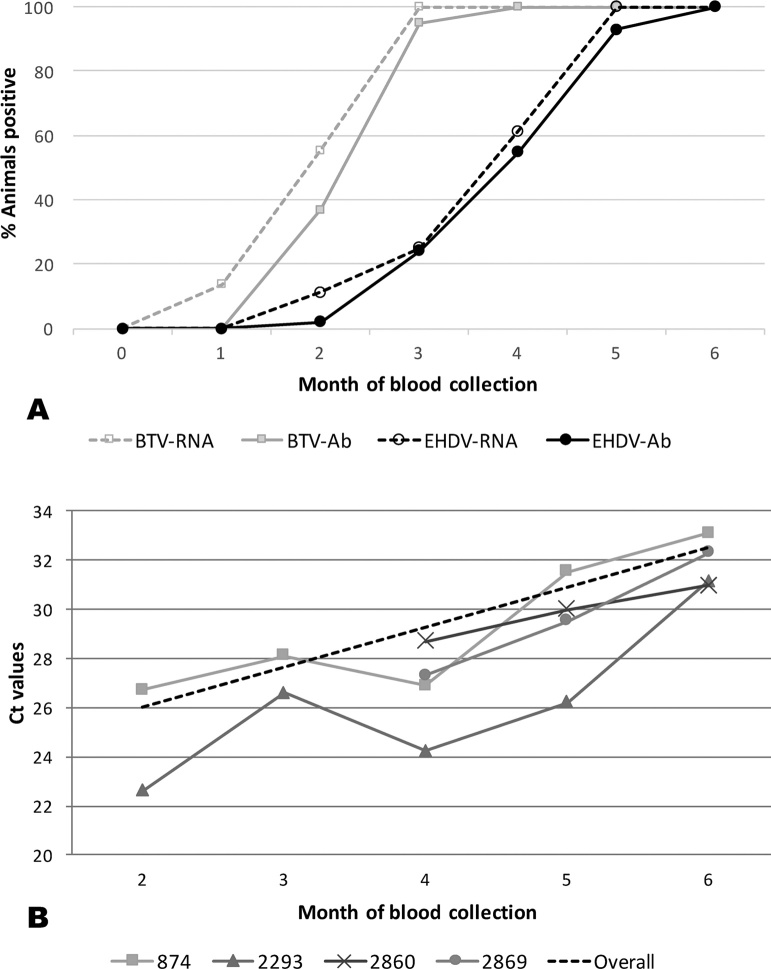
**A:** Percentage of cattle positive for antibodies (Ab) and RNA for both EHDV and BTV time of arrival in Trinidad (month 0) to the last month of blood collection (month 6).(BTV Ct values from their were taken from Brown-Joseph et al., 2017). **B:** Ct values measured by EHDV group-speciﬁc rRT-PCRs on blood samples taken from the ﬁrst month of virus detection to the last month of blood collection (month 6). The dotted line shows the overall trending increase in Ct values for animal 874 during the five-month period.

Fifty (83%) of the dairy cattle imported from the USA tested negative for EHDV antibodies three days after their arrival in Trinidad (month zero). No new seroconversions occurred in month one, one cow seroconverted in month two, eleven cattle seroconverted in month three, a further six cattle seroconverted in month four, nine cattle seroconverted in month five and the remaining three cattle seroconverted in month six. The time period to seroconversion for EHDV was longer than for BTV (data taken from Brown-Joseph et al., 2017) in the same cohort of cattle (Fig. 1). No clinical signs of EHD were observed in the infected animals. Throughout the six month study period a significant percentage of the cattle became unavailable for bleeding.

### 3.2 EHDV RNA levels gradually decreased over the six-month sampling period

None of the 60 cattle tested positive for EHDV-RNA upon arrival (month 0), nor in month 1. Six animals tested positive in month 2, a further four in month 3, nine in month 4, ten in month 5 and three in month 6. Of the ten animals that were seropositive for EHDV upon arrival, four were available for sampling for the entire six month period and tested positive for viral RNA for the first-time in months one, four, five and six.

In order to establish the kinetics of infection over the six-month study period, the Ct values from the group-specific rRT-PCRs were plotted for four animals that were available to sample for the entire six-month period and tested positive for EHDV-RNA by rRT-PCR for at least three consecutive months. Two of these selected animals were positive from month two and two became positive from month four. Overall, a gradual increase in Ct values over time was observed in these cattle (Fig. 1), corresponding to decreasing EHDV-RNA levels.

### 3.3 EHDV-6 identified by conventional RT-PCR/DNA sequencing, serotype-specific rRT-PCR and virus isolation

Thirteen of the selected (first-time) positive EHDV samples with Ct values ranging from 22.3 to 35.4 from the group-specific rRT-PCR tested positive for Group C in the conventional PCR EHDV-serotyping assay, which indicated that these samples contained viral RNA for either EHDV-6 or EHDV-8. The remaining nine samples tested negative for all four subgroups (A–D). The amplified (367 bp) band for Group C was subsequently sequenced, submitted to a megablast search and identified as EHDV-6 after being trimmed down to a 332 bp sequence (GenBank accession no. MH536521). Fourteen samples with the lowest Ct values (<30) in the EHDV group-specific rRT-PCR tested positive for EHDV-6 by serotype-specific EHDV rRT-PCRs. EHDV-6 was successfully isolated from one of these blood samples which was collected from cow ID# 2293 in month 2 after arrival in Trinidad. This isolate was deposited in The Pirbright Institute, Orbivirus Reference Collection, isolate reference number TAT2013/02.

### 3.4 Phylogenetic analysis of Trinidad EHDV-6 segment 2 (VP2) gene sequence

**Fig. 2:**
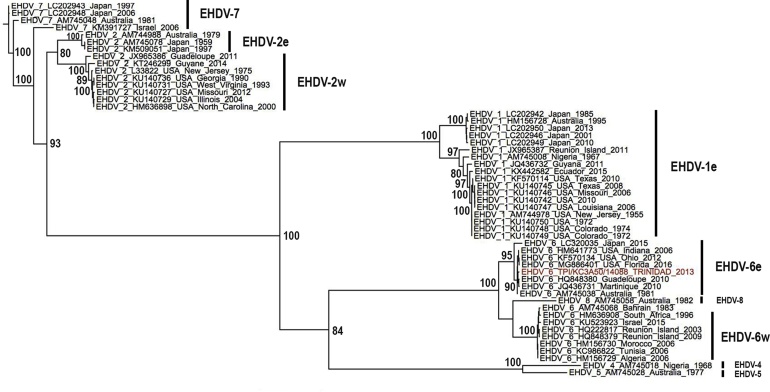
Maximum Likelihood (ML) tree estimated from 52 EHDV segment-2 sequences using PhyML with GTR + G_4_+I nucleotide substitution model. Taxon labels include year of isolation, accession number/ sequence ID and country of isolation. Clades defining eastern (e) and western (w) topotypes of EHDV serotypes are indicated by black bars. The Trinidad EHDV-6 sequence derived during this study is highlighted in red. Bootstrap support >80% is indicated at relevant nodes. (For interpretation of the references to colour in this figure legend, the reader is referred to the web version of this article).

A maximum likelihood tree was constructed from the data set of 52 EHDV VP2 sequences from Genbank including the Trinidad sequence from this study (Fig. 2). The sequences clustered into clades according to serotype with 100% bootstrap support for serotype 1, 2, 4–7 and with the eastern and western subtypes forming distinct subclades within their respective serotypes. The Trinidad sequence fell into the eastern topotype within the serotype 6 clade where it clustered with other Caribbean sequences.

Pairwise distances across all serotype 6 VP2 sequences ranged from 71.6 to 100% (Table S2) and were between 88.3 and 99.4% for the EHDV-6 eastern topotype to which the Trinidad sequences belonged. Nucleotide sequence identities between the Trinidad sequence and the sequences from the French Caribbean islands of Guadeloupe and Martinique were 97.1% and 96.8%, respectively. The Trinidad sequences also showed a sequence identity of 97.2% with the prototype Australian 1981 strain and sequence identities with USA strains (from Florida, Indiana and Ohio) ranged from 96% to 96.6%.

**Fig. 3:**
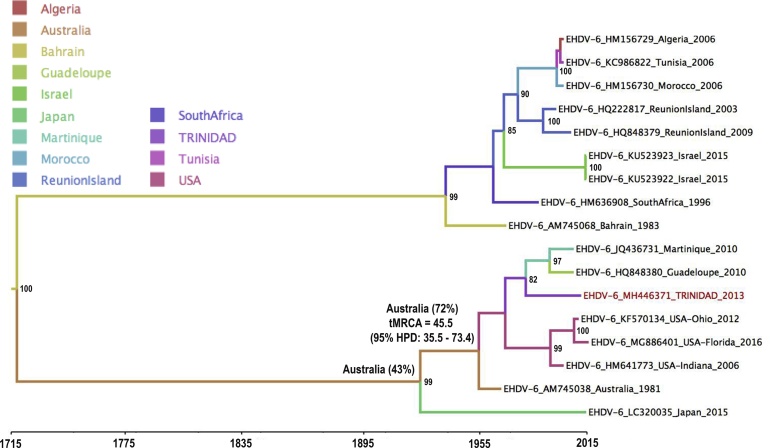
Maximum Clade Credibility (MCC) tree estimated from 17 EHDV-6 VP2 sequences (2916 bp). Taxon labels include accession number, country of isolation and year of isolation. Posterior probabilities (clade credibilities) >80% are indicated to the right of the relevant node. Terminal branches of the tree are coloured according to the country from which the sequence at the sequence was derived. Internal branches are coloured according to the most probable location of their parental nodes. The location state probability and estimated tMRCA for nodes basal to the clade containing the Caribbean and American strains is indicated as a percentage to the left of those nodes.

The Bayesian coalescent analysis of EHDV6 VP2 sequences (n = 17, 2916 bp each) is shown in Fig. 3. The mean estimate for when EHDV-6 VP2 sequences circulating in the Americas and Caribbean and the 1981 Australian prototype strain diverged was about 48.5 years (95% HPD 35.5–73.4 years) prior to 2015 and there is strong support that the most recent common ancestor (MRCA) for these two lineages existed in Australia (location state probability = 72%). Australia was also estimated to be the most probable location for the MCRA of all of the VP2 eastern topotype (location state probability = 43%) and that this ancestor existed about 93.9 years ago (95% HPD 35–224.3 years). The estimated overall mean rate of evolution for EHDV-6 VP2 was 2.7 × 10^−3^substitutions / site / year (95% HPD 1.7 × 10^-4^– 6.2 × 10^−3^ substitutions / site / year).

## 4 Discussion

Since 2009, Trinidad and Tobago (TTO) has been considered the Caribbean region’s leading dairy producer, despite the drastic decline in consumption (∼50%) of locally produced milk and milk products due to the importation of milk powder from North America, Europe and New Zealand (Agritrade, 2012). To help improve genetic stock and increase local meat and dairy productivity, TTO periodically imports dairy cattle from the USA. In June 2013 the government of TTO imported 60 dairy cattle from the USA to improve genetic livestock. This provided an opportunity to investigate the endemicity of *Culicoides*-borne Orbiviruses in Trinidad, particularly those that affect livestock trade, such as BTV and EHDV (Brown-Joseph et al., 2017; Verdezoto et al., 2017). Although EHDV infections in cattle are typically asymptomatic, certain EHDV serotypes (2, 6 and 7) have caused serious clinical disease in domestic cattle (Cêtre-Sossah et al., 2014; Allison et al., 2010; Temizel et al., 2009; Anthony et al., 2009; Yadin et al., 2008; Gaydos et al., 2004; Bréard et al., 2004), which can impact beef and milk production (CFSPH, 2016; Kedmi et al., 2010).

This study revealed that 17% (n = 10) of the imported dairy cattle had EHDV group-specific antibodies present in their blood three days after their arrival in Trinidad. Experimental studies have previously established that infectious EHDV-6 can be detected in cattle blood as early as 2–3 dpi and can persist for up to 28 dpi (Batten et al., 2011), and that EHDV RNA can remain for up to 50 days in the blood of both experimentally and naturally infected cattle (Aradaib et al., 1994; Abdy et al., 1999; Bréard et al., 2013). Therefore, the combination of positive antibody and negative rRT-PCR results in the 10 cattle three days after their arrival in the country indicated that they were not infectious upon entry into the country and that they were likely to have been exposed to EHDV some time ago while in the USA. Interestingly, four of the cattle that were seropositive on entry into Trinidad became positive for EHDV RNA within six months of arrival, indicating that they were most likely infected with an EHDV serotype in Trinidad different from the one they were infected with in the USA. Considering they were infected with EHDV- 6 in Trinidad, it is more likely that these cattle had been previously infected with either EHDV-1 or EHDV-2 while in the USA.

All of the naïve cattle seroconverted over the six-month period, with the peak number of seroconversions (n = 11) occurring in month 3. After initial infection, Ct levels slowly increased over the six-month study period (corresponding to decreasing EHDV-RNA levels), indicating that a single EHDV serotype was likely to be circulating in Trinidad, as was confirmed by the sole identification of EHDV-6 in the cattle. This was in contrast to the kinetics of infection observed for BTV in the same cohort of cattle over the same time period, where multiple serotypes of BTV were circulating in the cattle (Brown-Joseph et al., 2017). Epizootic haemorrhagic disease virus antibodies appeared in the cattle over a longer time period, with BTV antibodies being first-detected in 100% of the cattle by month three, as opposed to by month six for EHDV. Peak numbers of BTV infections (n = 21) occurred as early as month two, as opposed to month five (n = 11) for EHDV (Fig. 1).

The different infection kinetics between EHDV and BTV may be simply due to different amounts of virus circulating. Only one EHDV serotype (EHDV-6) was identified to be circulating in the cattle, whereas up to six different BTV serotypes were found to be co-circulating in the same cattle (Brown-Joseph et al. 2017). The differences observed in infection kinetics may also be due to differences in the abundance and competence of *Culicoides* vector species present in the area. *Culicoides insignis* Lutz 1913 is considered the putative vector for BTV in Southern USA, South America and the Caribbean (Tanya et al., 1992; Mo et al., 1994). The vast majority (∼94%) of the *Culicoides* captured near the sampled cattle were *C. insignis*, while other putative vector species, such as *C. pusillus* Lutz, 1913 represented ≤ 1% of the collected specimens (Brown-Joseph et al. 2017). Currently no *Culicoides* species has been formally identified as the putative vector for EHDV in the Caribbean, so it is possible that one of the less abundant *Culicoides* species, such as *C. pusillus,* may be a more competent vector for EHDV than the more abundant *C. insignis*, which would likely affect rates of transmission.

Phylogenetic analysis of the Trinidad EHDV-6 segment 2 (VP2) sequences, together with other VP2 sequences available in GenBank, showed that the Trinidad EHDV-6 segment-2 sequence belonged to the EHDV-6 eastern topotype and was most closely related to EHDV-6 viruses found in Guadeloupe, Martinique and the USA, with 96–97.2% nucleotide sequence identity. These levels of sequence identity, combined with data from the Maximum Clade Credibility (MCC) tree (Fig. 3), indicate that the Trinidad EHDV-6 VP2 sequence is phylogenetically distinct from its Caribbean neighbours (Martinique and Guadeloupe), despite their geographic proximity, and that some degree of evolution has occurred within the EHDV-6 VP2 sequences circulating in the Caribbean Basin. The data also provide statistical support for the hypothesis that EHDV-6 VP2 segments circulating in the Americas arose from an Australian ancestor which was estimated in this study to have existed in 1966 (95% HPD1941 and 1979). Comparison of the estimated overall mean rates of evolution for the VP2 segment between EHDV (2.7 × 10^−3^(nucleotide)substitutions / site / year (95% HPD 1.7 × 10^-4^–6.2 × 10^−3^ substitutions / site / year) and BTV (2.79 × 10^-4^substitutions / site / year (95% HPD 1.83–3.76 × 10^-4^substitutions / site / year) (Carpi et al., 2010), suggests that EHDV is evolving at a 10 fold faster rate than BTV.

Although, to date, it is not known which segment(s) of the EHDV genome is/are directly implicated in the virulence of the virus in cattle, evidence of severe clinical outbreaks in cattle infected with western topotypes of EHDV-6 in Morocco, Algeria and Turkey (Temizel et al., 2009; Kedmi et al., 2010) and a lack of clinical signs observed in cattle infected with eastern topotypes of EHDV-6 in the Caribbean and the USA, point towards a possible variation in virulence of EHDV related to its topotype. In order to further investigate this observation and to confirm whether the Trinidad EHDV-6 isolate is similar to the EHDV-6 strain circulating in the USA, which is a reassortant between the Australian EHDV-6 (CSIRO 753) and a local EHDV-2 USA Alberta parental strains (Allison et al., 2012), or whether it is a novel reassortant strain (that shares segment 2), full genome sequencing of the Trinidad EHDV-6 isolate is required. Additionally, experimental transmission studies using different species of *Culicoides* midges from different parts of the world are required in order to understand which species of *Culicoides* midges may vector the virus and whether this virus poses a risk of emergence in European countries.

## 5 Conclusion

This study revealed that EHDV-6 is circulating in domestic cattle in Trinidad, although the virus does not appear to be causing any clinical signs in infected cattle. The virus may however pose a severe risk to the large Red Brocket deer (*Mazama americana*) population in Trinidad. The Trinidad EHDV-6 (VP-2) was closely related to EHDV-6 viruses from the Caribbean, USA and the Australian EHDV-6 prototype strain, classifying it within the eastern topotype clade. Bayesian coalescent analysis supported Australia as the most probable source of the virus. Comparison of the estimated overall mean rates of evolution for the VP2 segment suggests that EHDV is evolving at a 10 fold faster rate than BTV. Further work, including *Culicoides* vector competence studies, along with full genome sequencing of the circulating strains, is necessary to elucidate the true impact of these findings.

## Conflicts of interest statement

The authors know of no ﬁnancial or personal conﬂicts of interest with any person or organization that could inappropriately inﬂuence this work. Funders had no role in study design nor the collection, analysis and interpretation of data.
