# Supplementary material for: Identification and characterization of epizootic hemorrhagic disease virus serotype 6 in cattle co-infected with bluetongue virus in Trinidad, West Indies
Source: Vet Microbiol. 2019 Feb;229:1–6. doi: 10.1016/j.vetmic.2018.12.009 (PMC6340808; doi:10.1016/j.vetmic.2018.12.009)
Supplement: Supplementary file 1 [file mmc1.docx]

**Table S1**List of nucleotide sequences used in this study and associated data.

| **No.** | **EHDV** | **Location** | **Accession No.** | **Year sample collected** | **Host species** | **Original sequence length (bp)** | **Manuscript Reference/**  **Date of Submission (d.s.)** |
| --- | --- | --- | --- | --- | --- | --- | --- |
|  |  |  |  |  |  |  |  |
|  |  |  |  |  |  |  |  |
|  |  |  |  |  |  |  |  |
|  |  |  |  |  |  |  |  |
| 1 | 1 | USA- New Jersey | AM744978 | 1955 | - | 2968 | Anthony et al., 2009 |
| 2 | 1 | Nigeria | AM745008 | 1967 | - | 2968 | Anthony et al., 2009 |
| 3 | 1 | Australia | HM156728 | 1995 | cattle | 2968 | Maan et al., 2010 |
| 4 | 1 | French Guiana (Guyane) | JQ436732 | 2011 | cattle | 2916 | Viarouge et al., 2014 |
| 5 | 1 | Reunion Island | JX965387 | 2011 | cattle | 2916 | Viarouge et al., 2014 |
| 6 | 1 | USA- Texas | KF570114 | 2010 | deer | 2916 | Anbalagan et al., 2014 |
| 7 | 1 | USA- Alabama | KU140742 | 2010 | deer | 2968 | Wilson et al., 2016 |
| 8 | 1 | USA- Texas | KU140745 | 2008 | deer | 2968 | Wilson et al., 2016 |
| 9 | 1 | USA- Missouri | KU140746 | 2006 | deer | 2968 | Wilson et al., 2016 |
| 10 | 1 | USA-Louisiana | KU140747 | 2006 | deer | 2968 | Wilson et al., 2016 |
| 11 | 1 | USA- Colorado | KU140748 | 1974 | deer | 2968 | Wilson et al., 2016 |
| 12 | 1 | USA- Colorado | KU140749 | 1972 | deer | 2968 | Wilson et al., 2016 |
| 13 | 1 | USA | KU140750 | 1972 | cattle | 2968 | Wilson et al., 2016 |
| 14 | 1 | Ecuador | KX442582 | 2015 | cattle | 2968 | Verdezoto et al., 2016 |
| 15 | 1 | Japan | LC202942 | 1985 | cattle | 2968 | Shirafuji et al., 2017 |
| 16 | 1 | Japan | LC202946 | 2001 | cattle | 2968 | Shirafuji et al., 2017 |
| 17 | 1 | Japan | LC202949 | 2010 | cattle | 2968 | Shirafuji et al., 2017 |
| 18 | 1 | Japan | LC202950 | 2013 | cattle | 2968 | Shirafuji et al., 2017 |
| 19 | 2 | Australia | AM744988 | 1979 | - | 3002 | Anthony et al., 2009 |
| 20 | 2 | Japan | AM745078 | 1959 | - | 3002 | Anthony et al., 2009 |
| 21 | 2 | USA- North Carolina | HM636898 | 2000 | deer | 3002 | Allison et al., 2012 |
| 22 | 2 | Guadeloupe | JX965386 | 2011 | cattle | 2949 | Viarouge et al., 2014 |
| 23 | 2 | Japan | KM509051 | 1997 | cattle | 3002 | Wu et al., (d.s.) 2014 |
| 24 | 2 | French Guiana (Guyane) | KT246299 | 2014 | cattle | 2949 | Viarouge et al., (d.s). 2015 |
| 25 | 2 | USA- Missouri | KU140727 | 2012 | deer | 3002 | Wilson et al., 2016 |
| 26 | 2 | USA- Illinois | KU140729 | 2004 | deer | 3002 | Wilson et al., 2016 |
| 27 | 2 | USA- West Virginia | KU140731 | 1993 | - | 3002 | Wilson et al., 2016 |
| 28 | 2 | USA- Georgia | KU140736 | 1990 | - | 3002 | Wilson et al., 2016 |
| 29 | 2 | USA- New Jersey | L33822 | 1975 | - | 3002 | Cheney et al., 1996 |
| 30 | 4 | Nigeria | AM745018 | 1968 |  | 3017 | Anthony et al., 2009 |
| 31 | 5 | Australia | AM745028 | 1977 | - | 3019 | Anthony et al., 2009 |
| 32 | 6 | Australia | AM745038* | 1981 | - | 2971 | Anthony et al., 2009 |
| 33 | 6 | Bahrain | AM745068* | 1983 | - | 2971 | Anthony et al., 2009 |
| 34 | 6 | Algeria | HM156729* | 2006 | cattle | 2971 | Maan et al., 2010 |
| 35 | 6 | Morocco | HM156730* | 2006 | cattle | 2971 | Maan et al., 2010 |
| 36 | 6 | South Africa | HM636908* | 1996 | cattle | 2971 | Anthony et al., 2009 |
| 37 | 6 | USA-Indiana | HM641773* | 2006 | deer | 2971 | Allison et al., 2012 |
| 38 | 6 | Reunion Island | HQ222817* | 2003 | cattle | 2919 | Sailleau et al., 2012 |
| 39 | 6 | Reunion Island | HQ848379* | 2009 | cattle | 2919 | Sailleau et al., 2012 |
| 40 | 6 | Guadeloupe | HQ848380* | 2010 | cattle | 2919 | Viarouge et al., 2014 |
| 41 | 6 | Martinique | JQ436731* | 2010 | cattle | 2919 | Viarouge et al., 2014 |
| 42 | 6 | Tunisia | KC986822* | 2006 | cattle | 2919 | Ben et al., 2016 |
| 43 | 6 | USA-Ohio | KF570134* | 2012 | deer | 2919 | Anbalagan et al., 2014 |
| 45 | 6 | Israel | KU523923* | 2015 | cattle | 2958 | Golender et al., 2017 |
| 46 | 6 | Japan | LC320035* | 2015 | cattle | 2919 | Kamomae et al., 2017 |
| 47 | 6 | USA-Florida | MG886401* | 2016 | deer | 2919 | Ahasan et al., 2018 |
| 48 | 6 | Trinidad | MH446371* | 2013 | cattle | 2971 | Brown-Joseph et al., (d.s.) 2018 |
| 49 | 6 | Trinidad | MH536521 | 2013 | cattle | 332 | Brown-Joseph et al., (d.s.) 2018 |
| 50 | 7 | Australia | AM745048 | 1981 | - | 3002 | Anthony et al., 2009 |
| 51 | 7 | Israel | KM391727 | 2006 | cattle | 3002 | Wilson et al., 2015 |
| 52 | 7 | Japan | LC202943 | 1997 | cattle | 3002 | Shirafuji et al., 2017 |
| 53 | 7 | Japan | LC202948 | 2006 | cattle | 3002 | Shirafuji et al., 2017 |

* Indicates the subset of (EHDV serotype 6) sequences selected for BEAST (v1.83) phylogeographic analyses.
